# A Baboon Brain Atlas for Magnetic Resonance Imaging and Positron Emission Tomography Image Analysis

**DOI:** 10.3389/fnana.2021.778769

**Published:** 2022-01-14

**Authors:** Artur Agaronyan, Raeyan Syed, Ryan Kim, Chao-Hsiung Hsu, Scott A. Love, Jacob M. Hooker, Alicia E. Reid, Paul C. Wang, Nobuyuki Ishibashi, Yeona Kang, Tsang-Wei Tu

**Affiliations:** ^1^Center for Neuroscience Research, Children’s National Hospital, Washington, DC, United States; ^2^Molecular Imaging Laboratory, Department of Radiology, Howard University, Washington, DC, United States; ^3^CNRS, IFCE, INRAE, Université de Tours, PRC, Nouzilly, France; ^4^Department of Radiology, Martinos Center, Boston, MA, United States; ^5^Department of Chemistry, Medgar Evers College, Brooklyn, NY, United States; ^6^Department of Electrical Engineering, Fu Jen Catholic University, New Taipei City, Taiwan; ^7^Department of Mathematics, Howard University, Washington, DC, United States; ^8^Department of Pediatrics, School of Medicine and Health Sciences, George Washington University, Washington, DC, United States

**Keywords:** baboon, brain, atlas, MRI, PET, imaging, registration

## Abstract

The olive baboon (*Papio anubis*) is phylogenetically proximal to humans. Investigation into the baboon brain has shed light on the function and organization of the human brain, as well as on the mechanistic insights of neurological disorders such as Alzheimer’s and Parkinson’s. Non-invasive brain imaging, including positron emission tomography (PET) and magnetic resonance imaging (MRI), are the primary outcome measures frequently used in baboon studies. PET functional imaging has long been used to study cerebral metabolic processes, though it lacks clear and reliable anatomical information. In contrast, MRI provides a clear definition of soft tissue with high resolution and contrast to distinguish brain pathology and anatomy, but lacks specific markers of neuroreceptors and/or neurometabolites. There is a need to create a brain atlas that combines the anatomical and functional/neurochemical data independently available from MRI and PET. For this purpose, a three-dimensional atlas of the olive baboon brain was developed to enable multimodal imaging analysis. The atlas was created on a population-representative template encompassing 89 baboon brains. The atlas defines 24 brain regions, including the thalamus, cerebral cortex, putamen, corpus callosum, and insula. The atlas was evaluated with four MRI images and 20 PET images employing the radiotracers for [^11^C]benzamide, [^11^C]metergoline, [^18^F]FAHA, and [^11^C]rolipram, with and without structural aids like [^18^F]flurodeoxyglycose images. The atlas-based analysis pipeline includes automated segmentation, registration, quantification of region volume, the volume of distribution, and standardized uptake value. Results showed that, in comparison to PET analysis utilizing the “gold standard” manual quantification by neuroscientists, the performance of the atlas-based analysis was at >80 and >70% agreement for MRI and PET, respectively. The atlas can serve as a foundation for further refinement, and incorporation into a high-throughput workflow of baboon PET and MRI data. The new atlas is freely available on the Figshare online repository (https://doi.org/10.6084/m9.figshare.16663339), and the template images are available from neuroImaging tools & resources collaboratory (NITRC) (https://www.nitrc.org/projects/haiko89/).

## Introduction

Baboons (*Papio anubis*) are primates phylogenetically close to humans with 96% of their genomes in common (Rogers et al., 2000; Comuzzie et al., 2003; Cox et al., 2013). The baboon and human brains share similarities in structure, function, and responses to neurological diseases, which make the baboon a popular laboratory primate species. Many neurobiology studies using baboons utilize imaging techniques such as PET or MRI for primary outcome measures (Horti et al., 2006, 2014, 2019; Szabó et al., 2007; Salinas et al., 2011; Griffith et al., 2012; Wu et al., 2014; Kumar et al., 2018). MRI can generate high-resolution images for evaluating brain structures (Wu et al., 2014) and provide matrices to identify neurodevelopmental disability (Griffith et al., 2012). PET is also widely used in baboon studies providing functional data for neurobiological processes *in vivo* (DeLorenzo et al., 2011). PET was used to investigate the role of neurotransmitter receptors using radioligands, such as [^11^C]JHU75528 for cannabinoid receptor system (Horti et al., 2006) and [^18^F]ASEM for α7-nicotinic cholinergic receptors in baboon brains (Horti et al., 2014). PET has been performed to study neuroinflammation (Horti et al., 2019), or to observe changes in cerebral blood flow with photosensitive epileptic baboons (Szabó et al., 2007), as well as to inspect disease models, including cancer (Kumar et al., 2018), atherosclerosis (Du et al., 2020), and Alzheimer’s (Tavitian et al., 1993) disease in baboons. Due to the fundamental limits of using scintillation detectors, PET demonstrates a clear drawback of low-resolution that makes it difficult to identify the borders between different brain areas. A brain atlas is an essential reference particularly important for PET to measure region-specific activity in the brain.

The Davis and Huffman histological atlas was the first atlas for baboon brains with sufficient details in the cerebral cortex areas (Davis et al., 1968). This photomicrographic baboon atlas did not provide a clear region definition and was, therefore, limited in its comprehensive application. The later Riche atlas provided anatomical labels on the histological brain sections in the orbito-meatal plane (Riche et al., 1988). This atlas was the first to be used in the analysis of PET images obtained from a benzodiazepine antagonist, [^11^C]Ro 15-1788, and a dopamine D2 receptor antagonist, [^76^Br]bromospiperone (Riche et al., 1988). The atlas referred to in Black et al. (2001) was a mapping of the Davis and Huffman atlas onto an MRI image template. The atlas’ regional information was created by a rigid alignment of the T1-weighted structural magnetic resonance template image and a PET blood flow template. These early baboon atlases approximated brain regions with labels in a photographic format, which makes them difficult to be utilized in a digital image analysis pipeline. In 2002, the Greer (Greer et al., 2002) atlas was developed based on baboon MRI images, and incorporated a similar focus as the current study to analyze PET images by regions. Like the Black atlas, the Greer atlas was developed on a 1.5-Tesla MRI image template and included a limited group of animals (*n* = 4–6) that confined its applicability and expandability to analyze current high-resolution image datasets. Compared to the extensive options available for the macaque brain atlas, existing baboon brain atlases are limited in their flexibility and usefulness in the modern baboon imaging study.

Recently, Love et al. (2016) performed a baboon brain template project to create a thorough and representative MRI image template from 89 individual baboons, including males, females, adolescents, and adults, to represent the brain anatomy of a large baboon population. This MRI template has 1.7 times greater resolution (0.6 mm^3^ voxel size) than the most recent published baboon brain template (0.49 × 0.49 × 1.5 mm^3^ voxel size) (Greer et al., 2002), allowing the development of a brain atlas with increased quality and accuracy (Black et al., 2001; Greer et al., 2002). The goal of this study was to create a baboon brain atlas based on the Love template to improve the analysis of PET and MRI data. Critical anatomical regions that are partially available in the previous atlases, including the corpus callosum, hippocampus, and amygdala, were delineated and incorporated into a cohesive single atlas for ease of unitary analysis. To assess the atlas as a viable tool for brain image analysis, atlas-based quantification results were compared with commonly-used PET and MRI measures, including PET time-activity curves (TAC), volumes of distribution (V_*T*_), standardized uptake values (SUV), and regions of interest (ROIs). The comparison measures were generated from manual segmentations developed by neuroimaging experts on both MRI and PET. Two sub-pipelines for PET image analysis were developed to test the atlas, depending on the availability of same-animal structural MRI images. Results suggested that the current baboon atlas generated from the group-averaged brain template facilitates an accurate and high throughput analysis of baboon PET and MRI images.

## Materials and Methods

### Image Data

The *in vivo* MRI template was obtained from the openly available Haiko89 Baboon Template Project (Love et al., 2016).^1^ Fifty-eight females and 31 males comprised the averaged image template, with ages ranging from 2.4 to 26.4 years (mean = 11.8 ± 6 years). A normal baboon’s life span is about 30 years, therefore, this template included baboons ranging from juveniles to older adults. The baboon PET data (*n* = 26) was acquired at Brookhaven National Laboratory from nine female baboons, ranging in weight from 13.5 to 21 kg, with five radioligands including [^18^F]flurodeoxyglycose (FDG) (Chen et al., 2021), [^11^C]benzamide (Seo et al., 2013, 2014), [^11^C]metergoline (Hooker et al., 2010), [^11^C]rolipram (Lourenco et al., 2001), and [^18^F]FAHA (Mukhopadhyay et al., 2006; Reid et al., 2009).

### Atlas Development

**FIGURE 1:**
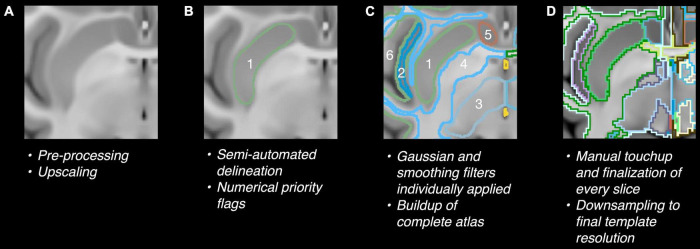
**(A)** 3X upscaling. **(B)** Manual segmentation and interpolation. **(C)** Combination of manually drawn labels and border linking. **(D)** Finalizing of label borders. Numbers in panels **(B,C)** indicate the priority flags of different brain regions.

The baboon’s anatomical structures were identified by referring to previously published histological and MRI atlases for baboon (Davis et al., 1968; Black et al., 2001; Greer et al., 2002). A diffusion MRI atlas for rhesus macaque (Calabrese et al., 2015) was used as a secondary reference to define a clear border between the regions that were only marked with labels but no clear regional definition in the existing baboon atlases, such as between the cerebral cortex, hippocampus, corpus callosum, and the cerebral white matter. The optimized automatic three-dimensional (3D) image registration toolbox from the MIPAV (NIH, Bethesda, MD, United States) software package was applied for the initial registration of the macaque atlas to the baboon template to create a rough visual outline of regions, utilizing affine registration with 12 degrees of freedom, nearest-neighbor interpolation, and the Powell’s calling Brent’s search algorithm (McAuliffe et al., 2001; Pluim et al., 2003). The atlas was constructed by a four-step procedure (Figure 1): (a) 3 × upscaling of the template image for finer delineation of an individual brain region, (b) The atlas was semi-automatically curated and delineated according to the T1-weighted contrast of the template using ITK-Snap (PICSL, Philadelphia, PA, United States) (Yushkevich et al., 2016). Regional segmentation was manually labeled on every 5th slice on the upscaled dataset as a starting point, and followed by inter-slice interpolation with careful manual modifications for in-between slices using the existing baboon and macaque atlases as references. Each region was given a priority rating based on its location relative to nearby regions and potential overlap with these regions upon the combination of labels. The lower priority labels were drawn slightly larger than the higher priority labels so that upon the combination, most labels would link seamlessly, (c) combination of regions using internally developed Matlab (Mathworks, Natick, MA, United States) scripts to merge every region border in the atlas. Gaussian filter (sigma = 2.0) and despeckle smoothing were applied to the combined region definitions for correcting disconnected pixels using FIJI (NIH, Bethesda, MD, United States) (Schindelin et al., 2012), (d) final manual touchup and downsampling to the native resolution of the template image.

### Atlas Evaluation for Magnetic Resonance Imaging

The atlas was validated by comparing to the expert’s hand-drawn delineation of ROIs on both MRI and PET per published protocols (Taha and Hanbury, 2015). A similar approach was applied to validate a macaque brain atlas with PET and MRI data (Moirano et al., 2019). In brief, four brains (#005, #020, #026, and #036) were randomly chosen from the 89 brains of the MRI template data pool, which are referred to as Subject I, II, III, and IV, respectively, in the validation test. Twenty four regions were manually segmented by two neuroscientists in four two-dimensional (2D) slices: two from the axial perspective, one from the sagittal, and one from the coronal. The manual and atlas-delineated ROI datasets were converted to 192 paired sets of binary 2D masks. The Dice Similarity Coefficient (DSC), also called the overlap index or Sørensen–Dice coefficient, was calculated for each ROI to quantify the agreement between the manual and atlas-based segmentation by:


(1) $$\begin{array}{c} D\,S\,C=\frac{2\,\left|X\,\cap Y\right|}{\left|X\right|+\left|Y\right|}=\frac{2\,T\,P}{2\,T\,P+F\,P+F\,N} \end{array}$$

Where *X* and *Y* are the cardinalities of the two ROI datasets. DSC is a statistical metric used to judge the geometric similarity of two ROIs (Zou et al., 2004; Taha and Hanbury, 2015), ranging from 0 (no overlap) to 1 (full overlap) (Dice, 1945). DSC > 0.7 is considered acceptable agreement (Bartko, 1991).

**FIGURE 2:**
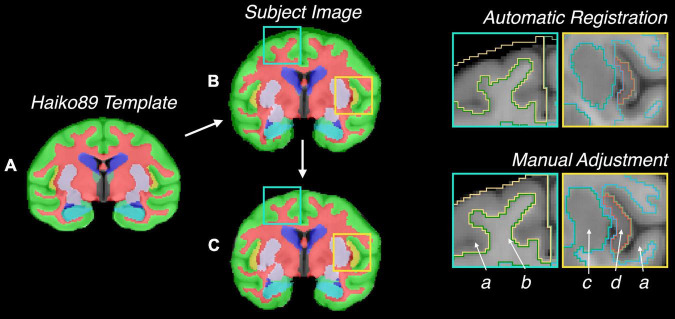
Illustration of semi-automatic registration for validation of the atlas for MRI data. **(A)** Atlas on Haiko89 template, **(B)** atlas transformed to Subject #036 by automatic registration, **(C)** final atlas registered to Subject #036 with additional manual adjustment. Highlighted squares indicate locations of automatic registration errors. Labels: (a) cerebral cortex, (b) cerebral white matter, (c) putamen, (d) insula.

The MRI subject datasets were registered to the atlas using the FMRIB Software Library’s FLIRT and FNIRT tools (Analysis Group, Oxford, United Kingdom) (Jenkinson and Smith, 2001; Jenkinson et al., 2002, 2012) by a 12-parameter affine model with the correlation ratio cost function (FLIRT) and the sum-of-squared differences cost-function (FNIRT), which is accepted as sufficient for most brains (Heinen et al., 2016; Bartel et al., 2019; Visser et al., 2020). A manual adjustment was then performed for registration errors that were found (Figure 2).

### Atlas Evaluation for Positron Emission Tomography

The atlas was tested for consistency and reproducibility in two PET image analysis pipelines, depending on the availability of same-animal structural images. Without an atlas, PET brain studies are in need of MRI or FDG based anatomical landmarks (Loessner et al., 1995; Hosaka et al., 2005), such as when acquired in dual-tracer PET experiments, to assist with registering images to a brain template on which the desired ROIs can be defined (PMOD Full Documentation, 2021). In the first pipeline, [^11^C]benzamide (Seo et al., 2013, 2014) (*n* = 3) PET data were analyzed, based on the same-animal FDG structural image to compare the manual segmentation and atlas-based analysis. To generate a reliable manual segmentation on FDG images, a human brain template available in PMOD (PMOD Full Documentation, 2021) (PMOD Technologies, Switzerland) was used for referencing the manual drawing of 12 brain regions, including caudate (left, right, and total), putamen (left, right, and total), thalamus (left, right, and total), and cerebellum (left, right, and total). Each [^11^C]benzamide PET image was then registered with rigid alignment to the same-animal FDG image to extract the regional TAC and V_*T*_ data. V_*T*_ was calculated by the Logan graphical method from the ratio of the radioligand concentration in the tissue target region to that of plasma at equilibrium (Logan et al., 1990; Innis et al., 2007). For the atlas-based analysis, each [^11^C]benzamide PET image was directly registered to the baboon atlas with SPM 8 (Wellcome Centre for Human Neuroimaging, London, United Kingdom) to extract the regional TAC and V_*T*_ of [^11^C]benzamide for comparison. These PET analysis methods were used in recent publications (Ikari et al., 2012; Zanderigo et al., 2018).

Secondly, the baboon atlas was evaluated for [^11^C]metergoline (Hooker et al., 2010) (*n* = 8), [^11^C]rolipram (Lourenco et al., 2001) (*n* = 6), and [^18^F]FAHA (Mukhopadhyay et al., 2006; Reid et al., 2009) (*n* = 3) PET data that had no same-animal anatomical images for structural reference. In this type of analysis, each PET image was registered to the atlas by 3D Slicer’s (NIH, Bethesda, MD, United States) landmark registration tool (Fedorov et al., 2012) with landmarks pre-defined in the putamen, caudate, cerebral cortex, and thalamus (Chollet et al., 2014). Eight brain regions were then manually traced on the [^11^C]metergoline, [^11^C]rolipram, and [^18^F]FAHA PET image slices centered in the mid-sagittal line and placed in the anterior-posterior commissure (AC-PC) orientation, including the thalamus, cerebral cortex, putamen, and cerebellum, each divided into left and right portions. Hand-drawn and atlas region datasets were converted to 272 paired sets of 2D masks. Each mask was associated with one predetermined slice from each dataset. As a result, eight [^11^C]metergoline, six [^11^C]rolipram, and three [^18^F]FAHA images, from two independent raters, constituted the datasets for comparison. DSC and intraclass correlation coefficients (ICC) were calculated for PET average SUV data per region to assess the reliability of atlas-based analysis. The ICC is defined by:


(2) $$\begin{array}{c} I\,C\,C=\frac{v\,a\,r\,\left(\beta \right)}{v\,a\,r\,\left(\alpha \right)+v\,a\,r\,\left(\beta \right)+v\,a\,r\,\left(\varepsilon \right)} \end{array}$$

where α is variability due to differences in the rating scales used by the judges, β is variability due to differences in the subjects, and ε is variability due to differences in evaluations by the judges (Bartko, 1966). ICC ranges from 0 to 1, where 0–0.5 represents a poor agreement, 0.5–0.75 is a moderate agreement, 0.75–0.90 is a good agreement and >0.90 is considered excellent agreement (Koo and Li, 2016).

### Statistical Analysis

The kinetic data, V_*T*_ and TAC, were organized into paired datasets, and a Pearson correlation was calculated for each with an accompanying *p*-value. DSC and ICC data were generated to indicate total variance between groups. Bland-Altman (BA) plots (Eastwood, 2001) and Kolmogorov-Smirnov (KS) tests for normality (Daniel, 1990, 319–330) were used to determine the distributions between hand-drawn and atlas-based SUV data with a 95% CI and significant level predetermined at *P* < 0.05. Paired SUV datasets were generated from the two judges for intra-rater and inter-rater analysis. All data are reported as mean ± standard deviation when appropriate. All analyses were performed with MATLAB programs of statistics toolbox (Mathworks, Natick, MA, United States).

## Results

### Atlas Development and Evaluation for Magnetic Resonance Imaging

**FIGURE 3:**
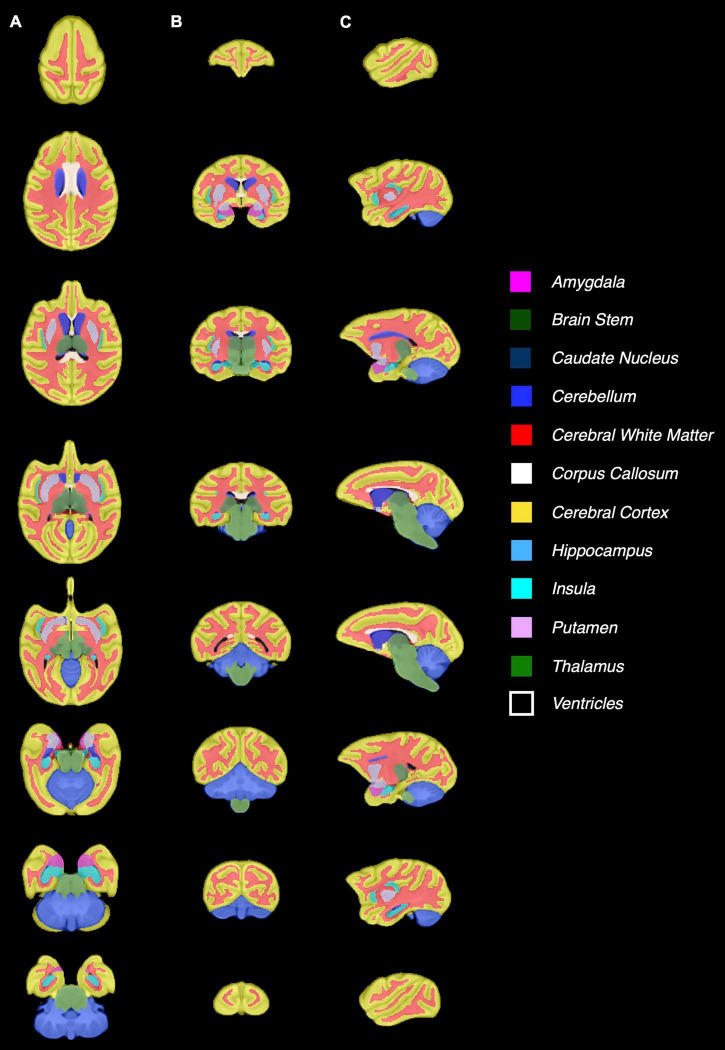
Demonstration of the baboon (*Papio anubis*) brain atlas. Nine different slices are presented for **(A)** axial, **(B)** coronal, **(C)** sagittal view.

**FIGURE 4:**
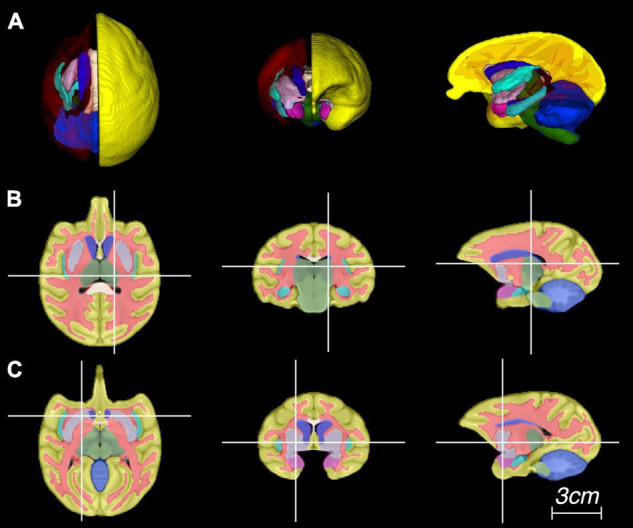
Representative images of the atlas in panel **(A)** three-dimensional rendering, and **(B,C)** multiplanar views centered at the thalamus and putamen, respectively.

Figure 3 shows the baboon brain atlas with 24 cerebral regions identified on the MRI template. Multiplanar views of the atlas at two different levels are illustrated in Figure 4. Table 1 lists the volume of each brain region in the atlas in comparison to that of four individual brains evaluated. The total brain volume of the atlas based on the Haiko89 template was 176.1 cm^3^, while that of the four individual brains ranged from 166.2 to 208.9 cm^3^ with a mean and standard deviation of 186.9 ± 18.2 cm^3^. The average DSC between atlas-based and manual segmentation was 0.83 ± 0.07 (Table 2). Smaller anatomical regions, such as caudate and insula, tended to show a lower DSC between the atlas-based and manual segmentation.

**TABLE 1:** Volume comparison of the brain regions identified in the atlas and from four individual brains (cm^3^).

**Region**	**Atlas**	**Subject I**	**Subject II**	**Subject III**	**Subject IV**	**Mean ± SD**
*Left cerebral cortex*	46.2	50.7	56.2	44.9	47.8	49.9 ± 4.8
*Right cerebral cortex*	46.0	52.2	55.9	44.5	48.1	50.2 ± 4.9
*Left caudate nucleus*	1.1	1.2	1.2	1.0	1.1	1.1 ± 0.1
*Right caudate nucleus*	1.2	1.2	1.2	1.0	1.2	1.2 ± 0.1
*Left hippocampus*	1.0	1.1	1.1	0.9	1.0	1.0 ± 0.1
*Right hippocampus*	0.9	1.0	1.0	0.8	1.0	1.0 ± 0.1
*Left cerebellum*	8.1	9.1	8.9	8.1	7.8	8.5 ± 0.6
*Right cerebellum*	8.2	10.0	9.5	8.4	8.1	9.0 ± 0.9
*Left lateral ventricle*	0.3	0.3	0.4	0.3	0.4	0.4 ± 0.1
*Right lateral ventricle*	0.4	0.4	0.4	0.3	0.4	0.4 ± 0.1
*Left amygdala*	0.5	0.6	0.5	0.5	0.5	0.5 ± 0.1
*Right amygdala*	0.5	0.6	0.5	0.5	0.5	0.5 ± 0.1
*Left brainstem*	4.1	4.3	4.4	3.6	3.9	4.1 ± 0.4
*Right brainstem*	3.9	4.1	4.1	3.4	3.8	3.9 ± 0.3
*Third ventricle*	0.1	0.1	0.1	0.1	0.1	0.1
*Left insula*	0.4	0.4	0.4	0.3	0.4	0.4 ± 0.1
*Right insula*	0.4	0.4	0.4	0.3	0.4	0.4 ± 0.1
*Left thalamus*	1.5	1.5	1.6	1.3	1.5	1.5 ± 0.1
*Right thalamus*	1.4	1.4	1.6	1.3	1.4	1.4 ± 0.1
*Left putamen*	1.9	2.0	2.1	1.7	2.0	2.0 ± 0.2
*Right putamen*	2.0	2.0	2.2	1.7	2.0	2.0 ± 0.2
*Left cerebral white matter*	22.3	23.5	26.9	20.1	22.5	23.2 ± 2.8
*Right cerebral white matter*	22.1	23.4	26.6	20.0	22.5	23.1 ± 2.7
*Corpus callosum*	1.4	1.4	1.7	1.2	1.4	1.4 ± 0.2
*Sum*	176.1	192.9	208.9	166.2	179.8	186.9 ± 18.2

**TABLE 2:** Atlas evaluation on T1-weighted magnetic resonance imaging (MRI) of four brain subjects by dice similarity coefficient between atlas and expert’s manual segmentation.

**Region**	**Subject I**	**Subject II**	**Subject III**	**Subject IV**	**Mean ± SD**
*Left cerebral cortex*	0.83 ± 0.03	0.88 ± 0.06	0.83 ± 0.04	0.86 ± 0.08	0.85 ± 0.02
*Right cerebral cortex*	0.80 ± 0.06	0.86 ± 0.07	0.81 ± 0.05	0.82 ± 0.05	0.83 ± 0.03
*Left caudate*	0.83 ± 0.20	0.82 ± 0.13	0.87 ± 0.14	0.80 ± 0.03	0.84 ± 0.03
*Right caudate*	0.84 ± 0.16	0.84 ± 0.10	0.88 ± 0.11	0.83 ± 0.03	0.85 ± 0.02
*Left hippocampus*	0.91 ± 0.07	0.85 ± 0.14	0.91 ± 0.08	0.88 ± 0.03	0.89 ± 0.03
*Right hippocampus*	0.88 ± 0.06	0.81 ± 0.15	0.85 ± 0.11	0.85 ± 0.10	0.85 ± 0.03
*Left cerebellum*	0.85 ± 0.02	0.95 ± 0.01	0.87 ± 0.05	0.94 ± 0.05	0.90 ± 0.05
*Right cerebellum*	0.84 ± 0.06	0.82 ± 0.03	0.87 ± 0.01	0.89 ± 0.04	0.86 ± 0.03
*Left lateral ventricle*	0.77 ± 0.08	0.73 ± 0.13	0.77 ± 0.08	0.76 ± 0.06	0.76 ± 0.02
*Right lateral ventricle*	0.75 ± 0.12	0.82 ± 0.25	0.76 ± 0.12	0.71 ± 0.03	0.76 ± 0.05
*Left amygdala*	0.83 ± 0.03	0.86 ± 0.08	0.78 ± 0.03	0.86 ± 0.13	0.84 ± 0.04
*Right amygdala*	0.88 ± 0.03	0.87 ± 0.13	0.87 ± 0.05	0.89 ± 0.08	0.88 ± 0.01
*Left brainstem*	0.87 ± 0.01	0.93 ± 0.03	0.88 ± 0.01	0.79 ± 0.13	0.87 ± 0.06
*Right brainstem*	0.88 ± 0.06	0.93 ± 0.03	0.91 ± 0.02	0.87 ± 0.03	0.90 ± 0.03
*Third ventricle*	0.76 ± 0.27	0.69 ± 0.23	0.71 ± 0.34	0.74 ± 0.28	0.73 ± 0.03
*Left insula*	0.70 ± 0.04	0.79 ± 0.11	0.68 ± 0.08	0.69 ± 0.08	0.72 ± 0.05
*Right insula*	0.71 ± 0.04	0.73 ± 0.20	0.66 ± 0.11	0.70 ± 0.16	0.70 ± 0.03
*Left thalamus*	0.91 ± 0.09	0.92 ± 0.02	0.92 ± 0.08	0.92 ± 0.07	0.92 ± 0.01
*Right thalamus*	0.87 ± 0.01	0.96 ± 0.03	0.88 ± 0.01	0.92 ± 0.03	0.91 ± 0.04
*Left putamen*	0.92 ± 0.05	0.91 ± 0.06	0.91 ± 0.07	0.91 ± 0.06	0.91 ± 0.01
*Right putamen*	0.80 ± 0.08	0.85 ± 0.09	0.81 ± 0.09	0.90 ± 0.04	0.84 ± 0.04
*Left cerebral white matter*	0.81 ± 0.06	0.84 ± 0.10	0.80 ± 0.08	0.81 ± 0.09	0.82 ± 0.02
*Right cerebral white matter*	0.81 ± 0.03	0.84 ± 0.09	0.77 ± 0.09	0.77 ± 0.08	0.80 ± 0.03
*Corpus callosum*	0.74 ± 0.03	0.69 ± 0.01	0.65 ± 0.02	0.65 ± 0.02	0.68 ± 0.05
*Mean ± SD*	0.83 ± 0.06	0.85 ± 0.08	0.82 ± 0.08	0.82 ± 0.08	0.83 ± 0.07

### Atlas Evaluation for Positron Emission Tomography Analysis

**FIGURE 5:**
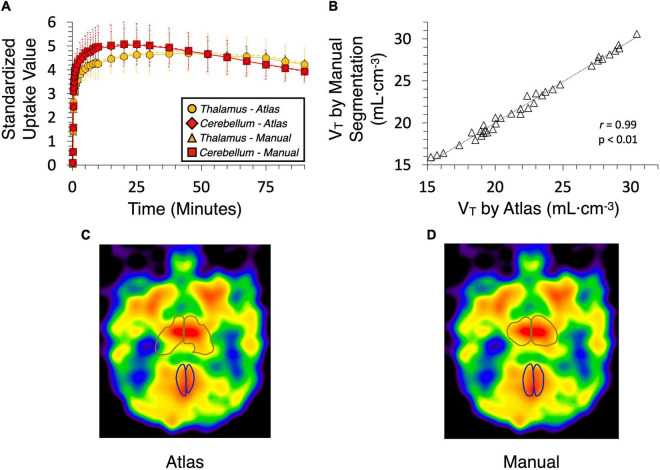
Time-activity curves (TAC) and volumes of distribution (V_*T*_) generated from dynamic [^11^C]benzamide data by baboon atlas and manual segmentation using the human brain template on PMOD. **(A)** TAC was obtained from the thalamus and cerebellum. **(B)** Correlation curve of V_*T*_ (*r* = 0.99, *P* < 0.01). **(C)** Thalamus and cerebellum are defined by the atlas. **(D)** Thalamus and cerebellum are defined manually.

**FIGURE 6:**
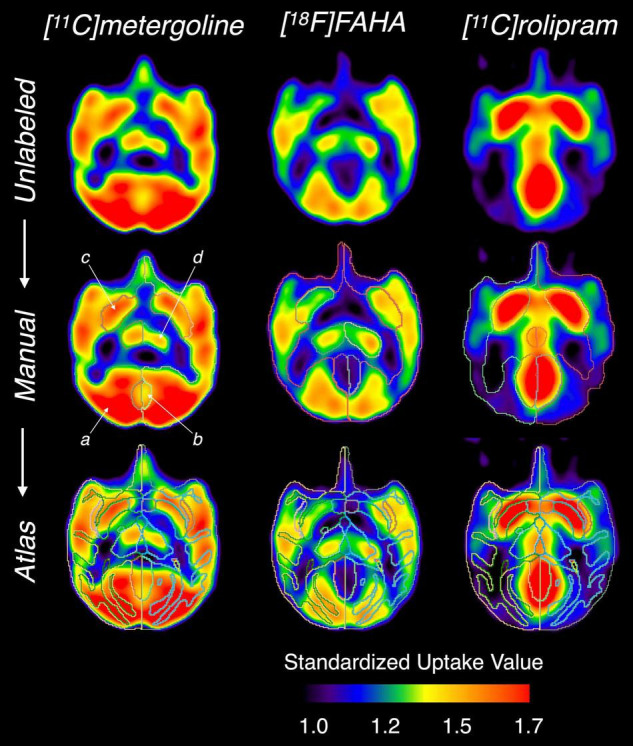
Comparison of the manual and atlas-based segmentation performed on different PET images. From the *top row*: unlabeled PET images, with manual segmentation, and with atlas-based segmentation. (a) cerebral cortex, (b) cerebellum, (c) putamen, (d) thalamus.

Results from analysis of dynamic [^11^C]benzamide PET data showed a high agreement of TACs and V_*T*_ (*r* = 0.99, *p* < 0.01) from every brain region measured, including cerebellum, thalamus, caudate, and putamen, between the manual and atlas-based quantification (Figure 5). In the analysis of static PET images, [^18^F]FAHA and [^11^C]rolipram images delivered high contrast in the gray matter regions, presenting clear identification of brain regions for both manual and atlas-based analysis (Figure 6). As a result, high ICCs (0.96 for both [^18^F]FAHA and [^11^C]rolipram) and moderate DSCs (0.58 ± 0.04 for [^18^F]FAHA; 0.62 ± 0.08 for [^11^C]rolipram) were seen. [^11^C]metergoline images showed high SUV in the gray matter regions, which resulted in the highest DSC agreement (0.64 ± 0.05) but with the lowest ICC at 0.81. The highest DSC (0.78 ± 0.07) was in the left thalamus of the [^11^C]rolipram PET, while the lowest DSC (0.38 ± 0.01) was displayed in the right thalamus of the [^18^F]FAHA PET (Table 3). For all three PET tracers, the highest ICC (0.97 ± 0.03) was found in the left cerebral cortex and the lowest (0.80 ± 0.14) was in the right cerebellum (Table 4). Compared to the MRI results, lower agreements were generally observed for PET between the atlas-based and expert-defined manual segmentation.

**TABLE 3:** Atlas evaluation on positron emission tomography (PET) images by dice similarity coefficient between the atlas and manual segmentation.

**Region**	**[^11^C]metergoline (*n* = 8)**	**[^18^F]FAHA (*n* = 3)**	**[^11^C]rolipram (*n* = 6)**
*Left putamen*	0.58 ± 0.15	0.74 ± 0.03	0.60 ± 0.22
*Right putamen*	0.54 ± 0.13	0.62 ± 0.09	0.57 ± 0.21
*Left cerebellum*	0.67 ± 0.14	0.62 ± 0.08	0.61 ± 0.15
*Right cerebellum*	0.62 ± 0.09	0.65 ± 0.01	0.61 ± 0.11
*Left thalamus*	0.70 ± 0.09	0.48 ± 0.01	0.78 ± 0.07
*Right thalamus*	0.66 ± 0.10	0.38 ± 0.01	0.68 ± 0.15
*Left cerebral cortex*	0.67 ± 0.04	0.63 ± 0.03	0.54 ± 0.09
*Right cerebral cortex*	0.67 ± 0.03	0.55 ± 0.05	0.54 ± 0.06
*Mean ± SD*	0.64 ± 0.05	0.58 ± 0.04	0.62 ± 0.08

**TABLE 4:** Atlas evaluation on PET images by the intraclass correlation coefficient between the atlas and manual segmentation.

**Region**	**[** ^11^ **C]metergoline (*n* = 8)**	**[** ^18^ **F]FAHA (*n* = 3)**	**[** ^11^ **C]rolipram (*n* = 6)**
*Left putamen*	0.90	0.96	0.98
*Right putamen*	0.79	0.97	0.95
*Left cerebellum*	0.70	0.98	0.92
*Right cerebellum*	0.64	0.92	0.84
*Left thalamus*	0.91	0.98	0.99
*Right thalamus*	0.93	0.94	0.99
*Left cerebral cortex*	0.93	0.99	0.97
*Right cerebral cortex*	0.85	0.99	0.96

**FIGURE 7:**
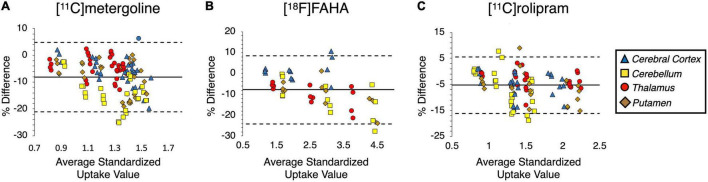
Bland-Altman plots of the SUV data acquired from **(A)** [^11^C]metergoline, **(B)** [^18^F]FAHA, **(C)** [^11^C]rolipram by an expert’s manual and atlas-based quantification. The solid line demonstrates mean SUV bias relative to manual quantification, and the dashed lines indicate 95% confidence of upper and lower bounds.

Results from the BA plots and KS tests showed that the percent differences in SUVs between manual and atlas rating were normally distributed for all tracers indicating the atlas-based analysis was not significantly biased relative to manual drawing (Figure 7). The atlas-based quantification had a bias for lower SUV than the manual quantification, with a mean bias for all tracers at −5.56 ± 3.36%. The highest bias appeared for [^11^C]metergoline PET at −8.17% and the lowest bias was for [^11^C]rolipram PET at −5.29%. High inter-judge agreements were found for all PET images, ranging from 0.97 ([^11^C]metergoline) to 0.99 ([^18^F]FAHA). The mean bias for the inter-judge agreement was −2.32 ± 1.64%. [^18^F]FAHA showed the highest inter-judge bias at −3.77%, and [^11^C]metergoline displayed the lowest at −2.23%. The inter-judge percent differences for [^11^C]rolipram PET were not normally distributed (*p* < 0.01, KS test), while that of the other two tracers appeared normally distributed (not significant, KS test). Intra-judge results showed excellent consistency of repeated drawings (Table 5). ICC ranged from 0.87 to 0.99 for both judges on all tracers, with a coefficient of variation ranging from 0.57 to 1.90%.

**TABLE 5:** Summary of atlas evaluation on PET.

**Atlas-judge**	**[** ^11^ **C]metergoline (*n* = 64)**	**[** ^18^ **F]FAHA (*n* = 24)**	**[** ^11^ **C]rolipram (*n* = 48)**
*Intraclass correlation coefficient*	0.81	0.96	0.96
*Dice similarity coefficient*	0.64 ± 0.05	0.58 ± 0.04	0.62 ± 0.08
*Bland-Altman Atlas Bias (%)*	−8.17	−7.86	−5.29
*Kolmogorov-Smirnov test of normality p*	0.06	0.94	0.36

**Inter-judge**	**[** ^11^ **C]metergoline (*n* = 64)**	**[** ^18^ **F]FAHA (*n* = 24)**	**[** ^11^ **C]rolipram (*n* = 48)**

*Intraclass correlation coefficient*	0.97	0.99	0.98
*Bland-Altman Judge A Bias (%)*	−2.23	−3.77	−3.22
*Kolmogorov-Smirnov Test of Normality p*	0.07	0.80	0.01

**Intra-judge (judge A/judge R)**	**[^11^C]metergoline (*n* = 4/*n* = 4)**	**[**^18^F**]FAHA (*n* = 8/*n* = 6)**	**[^11^C]rolipram (*n* = 4/*n* = 4)**

*Intraclass correlation coefficient (Judge A/Judge R)*	0.87/0.98	0.99/0.99	0.98/0.91
*CV (%) (judge A/judge R)*	1.24/0.68	1.06/0.57	1.00/1.90

## Discussion

Since there had been no fully segmented atlas available for the baboon brain, a new brain atlas was created to facilitate high throughput analysis of baboon imaging data. This atlas provides identification of 24 distinct brain regions based on the MRI template in a resolution that is at least 1.7 times higher than the existing atlases. The high-quality MRI template includes a large number of baboons of different ages, gender, and weight, which is more representative of a wider range of baboon brain structures (Love et al., 2016). The new atlas has been statistically evaluated not only with individual T1-weighted MRI but also with brain PET data generated using five known radiotracers. Results suggest that the current atlas is sufficiently well-representative of general baboon brain structure to benefit a high-throughput image analysis for PET and MRI.

An atlas is an important reference and tool for analyzing brain image data. It can facilitate analysis such as volumetric analysis, measuring regional uptake, and tracing dynamic responses with time (Gholipour et al., 2017). For example, in Parkinson’s disease volume changes of substantia nigra regions have been used to indicate disease progression and predict neurological disability (Ziegler et al., 2013). A well-defined brain atlas accompanied with automated registration, segmentation and quantification can replace arduous hand-drawing and expedite imaging analysis (Choi et al., 2016). This is particularly evident when dealing with large datasets that make manual segmentation impractical and human operator bias unavoidable. The atlas is especially helpful for analyzing PET data acquired without accompanying structural images to easily identify patterns of radiotracer distribution in large datasets (Bohm et al., 1986). The segmented standard atlas is also beneficial when analyzing images acquired by modern PET-CT or PET-MRI machines because it can be registered easily to anatomical CT or MRI images and provide detailed regional references (Judenhofer et al., 2007; Hofmann et al., 2008, 2009, 2011; Ruan et al., 2020).

Comparison of the manual and atlas-based image quantification showed consistently high agreements for both PET and MRI data (Table 5). High-resolution MRI was advantageous for performing registration in the atlas-based analysis to return a high DSC level (0.83 ± 0.07). The atlas was also tested on the analysis of PET images acquired with five different radioligands. Depending on the brain pharmacokinetics of the radiotracers, PET images can display variable contrast in different functional regions and usually do not present a clear anatomical structure of the brain. The BA plots showed that the atlas-based and the manual SUV values were comparable with a bias ranging from −5.29 to −8.17% depending on the tracer (Figure 7). In comparison, inter-judge agreements of the manual data appeared slightly tighter, ranging from −2.23 to −3.77% (Table 5). These results are similar to the data evaluated from the established atlases for other primates such as macaque (Gholipour et al., 2017).

Compared to the high DSC value (∼0.8) between the manual and atlas-based analysis in MRI, the DSCs were much lower in the PET images (0.58−0.64) (Table 3). This discrepancy may have been caused by insufficient PET image resolution that blurred the structural borders for a good registration and ROI delineation. Low-resolution PET images can also introduce a partial volume effect that spills image contrast over different regions of the brain (Gonzalez-Escamilla et al., 2021). Figure 6 shows the characteristic of different PET tracers that creates difficulty to identify the anatomy of activity-based geometry in the corresponding PET images. This issue may be resolved by acquiring simultaneous PET-MRI for low-uptake tracers or increasing the quality of registration between the MRI atlas and PET with future software packages.

High uptake tracers like [^11^C]metergoline delivered larger differences in SUV values, particularly in smaller regions, negatively influencing ICC. In the regions with low radiotracer uptake, the ICC value artificially increased due to the limitations of image contrast from PET of radiotracer activity. When radiotracer uptake decreased, the dynamic range of the SUV data lowered, and the ability to discern small changes decreased due to low contrast. Then there may appear to be higher ICC, while DSC and correlation remain lower than is suggested solely by the ICC data. To fully discern how much tracer uptake is present, both ICC and DSC data must be considered.

Without clear landmarks on PET images, manually segmenting presented unique challenges and was dependent on each expert’s own judgment. Confronting this limitation, the atlas-based analysis may alleviate such rater bias with a standardized process of registration to structural PET or MRI, associating the contrast in multiple brain regions and landmarks throughout the entire brain. The overall quality of atlas-based analysis may be higher than one rater’s manual segmentation since manual ROIs are usually drawn on one slice of the image volume at a time. For example, in [^11^C]rolipram PET, there was no clear border to identify the anatomical cerebral cortex region. As a result, the cerebral cortex on [^11^C]rolipram had the lowest DSC rating. In the absence of clear anatomical markers, the atlas may provide a standard reference to compare uptake values from the blurry low contrast PET images, as is done in PMOD (PMOD Full Documentation, 2021). Further confounding the issue, disagreement between judges was not consistent while the atlas-based segmentation and expert drawings consistently disagreed on specific regions with all tracers, demonstrating the consistency of atlas-based analysis. As a result, the average bias between judges was lower (3.07 ± 0.78%) than that for judge-atlas (7.11 ± 1.58%).

Comprehensive labeling of every neuroanatomic structure in the Haiko89 MRI template has yet to be done. The current baboon atlas provides definitions of 24 distinct brain regions sufficient for most PET image analyses. To account for acute deformities in brains, secondary atlas may be required. For example, human brains that have been significantly affected by Alzheimer’s disease will have clear imaging biomarkers such as abnormalities in the hippocampus and amygdala, and ventricular enlargement in a U-shape (Coupé et al., 2019). Since these changes are very stark in comparison to a healthy brain, a secondary atlas could increase the quality of registration and subsequent quality of segmentation. The current atlas can also serve as a foundation for the future development of more detailed atlases. This primary baboon atlas may be transformed onto a particular experimental dataset, and manually modified as necessary to create a secondary atlas to be used in conjunction with the primary. The future secondary atlas would serve to address the limitations of a single atlas when used with particular research demands. Since the current atlas was created based on the Haiko89 template image that incorporated all of the sub-groups together, a secondary atlas is needed to provide substantial benefit for analysis accuracy when used with datasets consisting of only specific subgroups such as elderly baboons, young baboons, or datasets with only one sex.

MRI scans that had profound artifacts were less likely to be registered successfully, adversely impacting the accuracy of segmentation. Similarly, another component of difficulty was registering the PET and MRI images. This was countered in this study by utilizing well-documented automated registration for MRI images and manual landmark registration between PET and MRI images (Neelin et al., 1993; Shan et al., 2011; Keszei et al., 2017). At the time of writing, there is an active research and proprietary development of automated solutions for the issue of independent PET-MRI image registration (Robertson et al., 2016). Future atlas-based analysis may be incorporated with automated registration solutions, allowing rapid analysis of the images acquired between different imaging modalities.

## Conclusion

This study describes the creation of a high-quality digital atlas for the baboon brain that will help combine PET functional/neurochemical data with MRI-based anatomy. The new atlas was demonstrated useful to acquire comparable results with the expert manual quantification of T1-weighted MRI and PET images acquired with five different radioligands. Since the baboon and human brain share large similarities in structure, function, and metabolism, an atlas-based approach in the baboon model may provide exceptional data needed to inform human neuroimaging studies. The atlas is made publicly available in electronic format to benefit the baboon research community that utilizes MRI and PET.

## Data Availability Statement

The datasets presented in this study can be found in online repositories. The name of the repository and accession number can be found below: Figshare, https://doi.org/10.6084/m9.figshare.16663339.

## Ethics Statement

The animal study was reviewed and approved by Institutional Animal Care and Use Committee (IACUC).

## Author Contributions

AA, T-WT, PW, YK, and NI conceived of the study and provided project structure, data, and direction. JH and AR provided PET data. RS assisted with atlas creation, background research collection, and carried out validation data creation tasks. RK assisted with MRI validation data. C-HH assisted with atlas modification scripts in MATLAB. SL provided MRI data and project direction feedback. All authors contributed to the article and approved the submitted version.

## Conflict of Interest

The authors declare that the research was conducted in the absence of any commercial or financial relationships that could be construed as a potential conflict of interest.

## Publisher’s Note

All claims expressed in this article are solely those of the authors and do not necessarily represent those of their affiliated organizations, or those of the publisher, the editors and the reviewers. Any product that may be evaluated in this article, or claim that may be made by its manufacturer, is not guaranteed or endorsed by the publisher.

## Footnotes

1
https://www.nitrc.org/projects/haiko89/
